# Patient compliance to postoperative instructions after third molar surgery comparing traditional verbally and written form versus the effect of a postoperative phone call follow-up a: A randomized clinical study

**DOI:** 10.4317/jced.56680

**Published:** 2020-10-01

**Authors:** Amparo Aloy-Prósper, Hilario Pellicer-Chover, José Balaguer-Martínez, Oscar Llamas-Monteagudo, Miguel Peñarrocha-Diago

**Affiliations:** 1 DDS, PhD. Assistant Professor of Oral Surgery, Stomatology Department, Faculty of Medicine and Dentistry, University of Valencia, Spain; 2 DDS, PhD. Collaborating Professor of the Master in Oral Surgery and Implant Dentistry, Stomatology Department, Faculty of Medicine and Dentistry, University of Valencia, Spain; 3 DDS, MSc. Student of Master in Oral Surgery and Implant Dentistry, Stomatology Department, Faculty of Medicine and Dentistry, University of Valencia, Spain; 4 Master in Oral Surgery and Implant Dentistry, Stomatology Department, Faculty of Medicine and Dentistry, University of Valencia, Spain; 5 MD, PhD. Chairman of Oral Surgery, Stomatology Department, Faculty of Medicine and Dentistry, University of Valencia, Spain

## Abstract

**Background:**

The understanding and adherence to postoperative care instructions may be influenced by how they are presented by the professional interfering the recuperation process after surgery. The aim of this study was to evaluate the effect of a postoperative phone call follow-up compared with a traditional verbally and written instructions regarding compliance of postoperative recommendations after third molar surgery; and secondly, to discover the main points of non-compliance.

**Material and Methods:**

A randomized clinical study was performed including patients that underwent surgical extraction of an impacted mandibular or maxillary third molar in the Oral Surgery Unit of the University of Valencia from January 2016 to January 2017. Patients were randomly assigned to one of three different test groups according to how the post-operative instructions were delivered: brief written instructions, written extended instructions or brief written instructions plus a phone call follow-up at 3-day postoperative period. Patients were interviewed about their adherence to the instructions one week after surgery. The significance level was set at *p*<0.05.

**Results:**

The higher score of compliance was found to the phone call follow-up group (*p*=0.001). No statistically significant differences were found between brief written group and the group that received written extended instructions. In the phone call follow-up group all variables assessed to the compliance were fulfilled. To brief written and written extended instructions groups, the main points of non-compliance were hygiene and smoking (*p*<0.001, *p*=0.026, respectively), and tended towards significance for chlorhexidine rinses and antibiotic, analgesic and anti-inflammatories medication prescribed.

**Conclusions:**

Telephone call follow-up can promote patient adherence to postoperative recommendations after third molar surgery. The main factors of non-compliance were not maintain a proper hygiene and not smoking, followed by not performing chlorhexidine rinses and not following medication prescribed.

** Key words:**Compliance, postoperative instructions, postoperative recommendations, third molar surgery.

## Introduction

Surgical removal of impacted third molars remains one of the most common procedures performed by oral and maxillofacial surgeons (1). The understanding and adherence to postoperative care instructions may be influenced by how they are presented by the professional (verbally and/or written) interfering the recuperation process after surgery (2).

Few reports are published regarding the surgeon’s explanations and patient comprehension and implementation of the instructions after oral surgical procedures (2-6), especially in third molar surgery literature is scarce (2,5). Some studies have shown that written instructions are a valuable supplement to verbal instructions in order to increase patient understanding (4,5). However, a clinical trial published by Alvira-González & Gay-Escoda (2) did not find differences in the adherence to postoperative instructions regarding how they were provided to the patient (verbal, written and a group that received written additional information). Atchison *et al.* (7) found that approximately 41% of patients recalled elements of postoperative instructions over postoperative period. Previous studies, although not dental reports, demonstrated that a telephone call follow-up is a safe and cost-effective method to ensure and maintain optimal patient outcomes (8-12). To the best of the authors’ knowledge, this is the first work that analyzes the benefit of a phone call follow-up to improve the compliance of postoperative recommendations in the field of dentistry. The hypothesis is that a phone call follow-up during the postoperative period will enhance the implantation of the instructions and address any questions or concerns after third molar surgery.

The main objective of this study was to evaluate the effect of a postoperative phone call follow-up compared with a traditional verbally and written instructions regarding compliance of postoperative recommendations after third molar surgery; and secondly, to discover the main points of non-compliance.

## Material and Methods

-Study design

A randomized clinical trial was performed including patients that underwent surgical extraction of an impacted mandibular or maxillary third molar in the Oral Surgery Unit of the University of Valencia from January 2016 to January 2017. The study was performed following the instructions of the Declaration of Helsinki for human research. Accordingly, all patients received information about the study, and were asked to sign a written informed consent form before taking part. The study design was approved by the Ethics Committee of the University of Valencia (Ref.: H1450093433934). The present study is presented in accordance with the CONSORT statement (13).

-Selection Criteria

The inclusion criteria were healthy patients, older than 18 years who agreed to participate in the study and signed an informed consent. The exclusion criteria were patients with mental conditions or psychological disorders that do not allow them to understand and implement the postoperative instructions, and those who could not attend the scheduled appointments.

-Randomization

Patients were randomly assigned to one of the following three study groups (sequence generated by a computer program (IBM SPSS v21; IBM Corp) macro (!RNDSEQ; Domenech JM, Granero R):

- Group 1. Brief written instructions: usual postoperative instructions were given verbal and on paper briefly (Table 1).

**Table 1:**
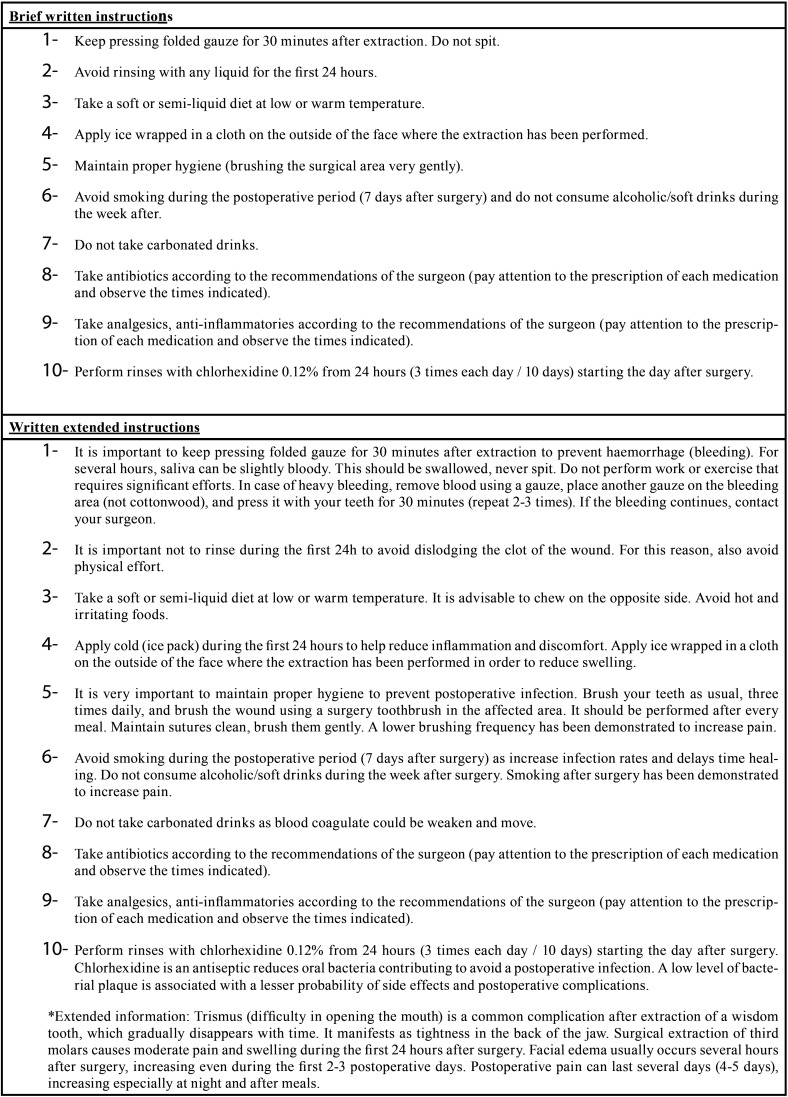
Brief and extended postoperative information provided after the third molar surgery.

- Group 2. Written extended instructions: instructions and postoperative medication were given both verbally and written, and extended written information about the postoperative period was also provided (Table 1).

- Group 3. Reinforcement phone call follow-up: instructions were given verbally and on paper briefly (as group 1), in addition patients were phoned at 3 day after surgery to ensure the compliance of recommendations.

Postoperative information provided was based on Alvira-Gonzalez & Gay-Escoda study (2). Patients did not receive any financial compensation for their participation in the study. All surgeons involved in the study were blinded, as well as the researcher and the statistician. The recommendations and recalls were delivered by a different clinician to surgeon.

-Procedure

The surgery was performed under local anesthesia with 4% articaine 1:100.000 adrenalin (Inibsa, Lliça of Vall). A vestibular triangular mucoperiosteal flap was raised with a distal incision and vestibular release. The osteotomy and odontectomy were made using a rounded tungsten carbide drill, mounted in a hand piece, with abundant irrigation of sterile physiologic serum. After extracting the molar, the cavity was inspected and sutured with 3-0 silk (Lorca Marin, TB15, 3/8, Murcia, Spain). All patients received postoperative antibiotic treatment: amoxicillin 500 mg, 3 times during 1 week. Ibuprofen 600 mg, 3 times daily during 4 days, and patients were instructed to rinse with 0.2% chlorhexidine. Sutures were removed one week after the surgery. All surgical procedures were carried out by surgeons with similar experience of the Master of Oral Surgery and Implantology of the University of Valencia. After the surgical procedure, postoperative recommendations were given according to the study groups.

-Data collected

At surgery, the following variables were recorded: age, gender, tooth to be extracted (relative position and arcade), frequency of brushing (≥ 3 times/day; 1-2 times/day) smoking habits (no smoking; < 10 cigarettes/day; 10-20 cigarettes/day; >20 cigarettes/day) and surgical difficulty using Pell&Gregory index (1933). The principal predictor variable was how the recommendations were given (brief written instructions, written extended instructions and a reinforcement postoperative phone-call).

The primary outcome variable of interest was the score of compliance of postoperative instructions. Patients were interviewed about their adherence to the instructions one week after the surgery, at the time of suture removal. They were requested about the compliment of the instructions (Table 2). For each patient, the final score was the sum of the scores for each question (maximum 10 points).

**Table 2:**
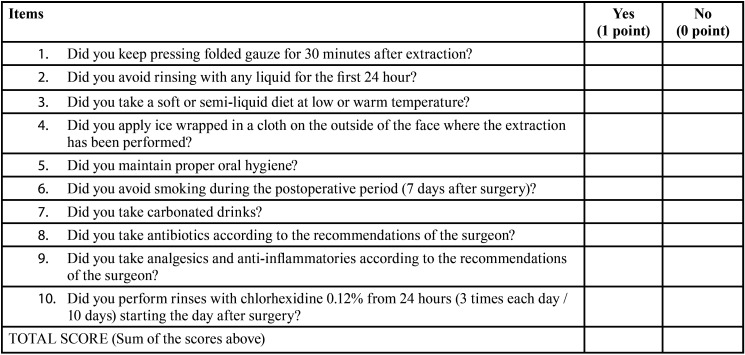
Questionnaire to assess the compliance of postoperative instructions following the third molar surgery.

-Statistical analysis

The collected data was tabulated and statistically evaluated. Frequency distribution and percentage analysis were done. Homogeneity between patient groups was analyzed using parametric tests in the quantitative variables, as them showed a normal distribution (Shapiro-Wilk and Kolmogorov-Smirnov tests *p*>0.05). Chi-square test was used to evaluate homogeneity regarding sex, hygiene, smoking habits and surgical difficulty, while the t-Student was used to evaluate homogeneity regarding age. ANOVA test was used to compare the compliance and how the postoperative instructions were provided (brief written, extended written instructions and postoperative phone call follow-up). A linear regression model was used to analyze the total score obtained in compliance regarding each of the parameters studied.

To our knowledge, there were no studies in the literature about the influence of a phone call in the adherence to the instructions provided to the patient. It was necessary to determine the size of the effect and calculate the sample size. An initial sample of 40 patients allocated for each group by simple randomization was determined. It was found a size effect of 1.38 (short group mean = 8.39, call group = 9.89, pooled SD = 1.08). To ensure a power of 90% capable of detecting an effect of 1 with a confidence of 95% and alpha set at 0.05, it was needed to include a minimum of 18 subjects of every group. An additional 20% of patients were included in the trial to prevent statistical power loss for attrition. Statistical analysis was completed using SPSS 21.0 software (SPSS Inc., Chicago, IL).

## Results

Seventy-seven patients underwent third molar surgery. Eight patients were excluded because they did not attend to the 7-day follow-up visit (2 brief written instructions group, 2 extended written instructions group, 4 phone-call follow-up group). The final study sample included 69 patients (34 women and 35 men) with an average age of 25.9 ± 9.2 years (range 18 to 45). Sample size and composition is shown in Table 3, with no statistically significant differences between groups.

**Table 3:**
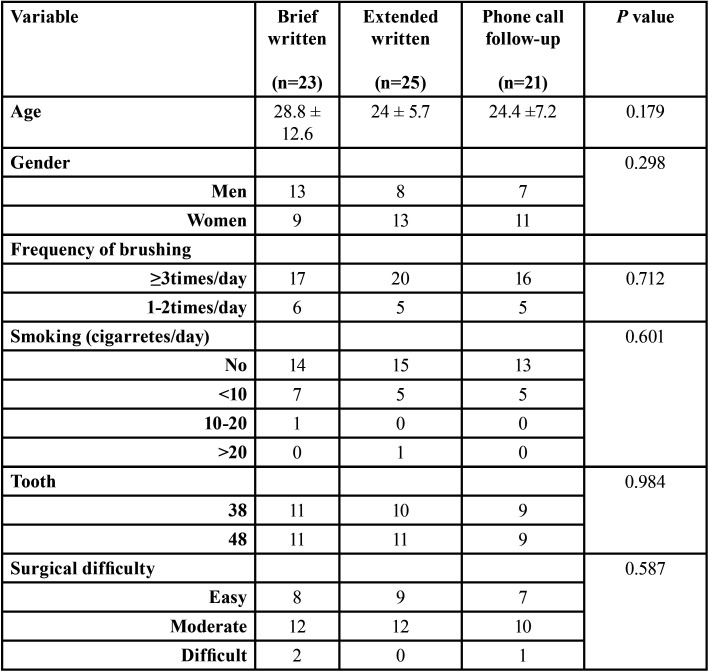
Description of the patient sample per group.

The higher score to the level of compliance was found to the phone call follow-up instructions group (9.7±0.5) in comparison to brief written instructions (8.5±1.2) and written extended instructions (7.9±2.1) groups, being this difference statistically significant (ANOVA *p*=0.001) (Table 3).

Regarding the variables most related to non-compliance, in the phone call follow-up instructions group all variables assessed were fulfilled; while in the brief written and extended written instructions groups, hygiene and smoking were the variables that were less fulfilled, being this differences statistically significant (*p*<0.001 and *p*=0.026, respectively), and tended towards significance for NSAID (*p*= 0.078), chlorhexidine rinses (*p*=0.075) and ATB (*p*=0.052); the written extended instructions group showed worse results than brief written instructions group (Table 4).

**Table 4:**
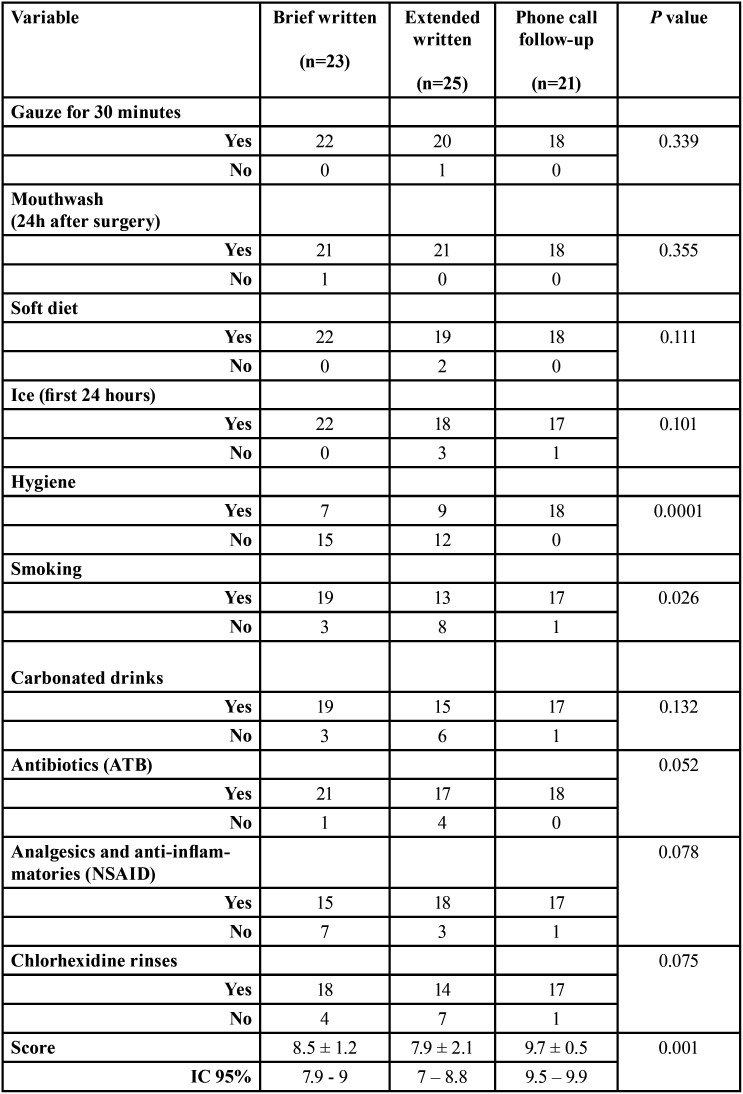
Relationship between compliance of each assessed variable and study groups.

Overall, after applying linear regression analysis, a very high goodness of fit (R2= 0.983) was observed. The most important factor to the non-compliance (score variable) was hygiene, with a variation of 25.2% (*p*= 0.0001), followed by smoking with 19.1% (*p*= 0.0001), chlorhexidine rinses 14.2% (*p*= 0.02), NSAID 13.3% (*p*= 0.01) and carbonated drinks 13.2% (*p*= 0.03).

## Discussion

A growing number of patients have difficulty to understand and implement postoperative instructions. Applying correctly the postoperative instructions after surgery reduce morbidity, help a fast recovery and improve the quality of life of patients. Schouten *et al.* (14) found that patients’ satisfaction was mainly influenced by the communicative behaviour of the dentist. Anxiety or stress that arises from a surgical procedure may detract the patient’s ability to concentrate on information surgeon is given after the surgical procedure (3,15). Alexander *et al.* (3) found that available written post-surgical procedures instructions in dentistry are replete with poor phrasing; excessive jargon and bad terminology, which may interfere to the compliance of postoperative recommendations. Layton *et al.* (16) showed that nearly half of patients undergoing third molar surgery failed to recall or recognize at least one of the preoperative verbal instructions given by the doctor. In addition, Vallerand *et al.* (5) demonstrated that postoperative pain control and satisfaction after third molar surgical extractions were greater in patients who received detailed recommendations and information. The way the information is delivered to patients plays a paramount role in the level of compliance of postoperative recommendations. Alexander *et al.* (3) showed that without written reinforcement the understanding and retention of verbal instructions over a lengthy period of recovery cannot be assured. Similar results were reported in the study by Atchison *et al.* (7) who showed that a combination of verbal and written instructions are preferred by most patients, particularly those with lower education. Alvira-Gonzalez & Gay-Escoda (2) did not find statistically significant differences regarding adherence of postoperative care instructions depending on the manner of instruction presentation (verbal, written, additional written instructions), preoperative anxiety level and sociocultural level. Telephone contact with patients has been demonstrated to be a useful tool in providing a means for questions and concerns to be addressed in the critical time for patients and families after surgery (9). A Cochrane review of postoperative phone call follow-ups conducted by various health care professionals for patients discharged from the hospital found that postoperative phone calls made by hospital-based health professionals was considered a good means of information exchange for symptom management, patient instruction and education and early recognition of potential complications (17). In the present study, all patients received verbal instructions; results showed that a reinforcement phone call follow-up at 3-day after surgery showed the best score in compliance of postoperative recommendations statistically significant. Moreover, no differences were found between the group that received brief written instructions and the group that received extended written instructions as Alvira-Gonzalez & Gay-Escoda (2) found.

Hygiene and smoking were the variables more related to the non-compliance with the postoperative recommendation, followed by not performing chlorhexidine rinses and NSAID and ATB prescription. Conrad *et al.* (18) found that food collection in the surgical sites posed the biggest problem for patients (20%). In the present study, a 25.2% of patients failed in maintaining a proper hygiene during the postoperative period. This might be due to some patients might experience pain and discomfort during brushing of surgical sites as well as ignorance or fear about how clean these areas. So the clinician should educate patients on oral hygiene instructions in order to gain confidence at the time of maintaining hygiene properly. Regarding smoking, over 19% of smokers continued smoking during the postoperative period. Sweet (19) and Meechan *et al.* (20) reported that the majority of patients who smoked continued also doing postoperatively. The use of chlorhexidine rinses, NSAID and consumption of carbonated drinks was failed by over 13% of patients. The main cause of abandonment was relief of pain considering they were able to return to their daily lives without caring on that.

Few data is available regarding the surgeon’s explanations and patient comprehension and compliance of postoperative instructions after the surgical extraction of third molars. Future studies are needed to support the results of this RCT to improve the level of communication between the surgeon and the patient.

Telephone call follow-up can promote patient adherence to postoperative recommendations after third molar surgery. The main factors of non-compliance were not maintain a proper hygiene and not smoking, followed by not performing chlorhexidine rinses and not following medication prescribed.
